# Environmental and occupational respiratory diseases – 1038. Efficacy of influenza vaccination on pediatric asthma control

**DOI:** 10.1186/1939-4551-6-S1-P37

**Published:** 2013-04-23

**Authors:** Mohammad Reza Fazlollahi, Azadeh Shaabani, Zahra Pourpak, Raheleh Dashti, Masoud Movahedi, Mohammad Gharagozlou, Asghar Aghamohamadi, Mostafa Moin

**Affiliations:** 1 Tehran University of Medical Sciences, Immunology,Asthma and Allergy Research Institute, Tehran, Iran; 2 Tehran University of Medical Sciences, Children’s Medical Center, Tehran, Iran

## Background

Influenza vaccination have been recommended for asthmatic patients in many countries as observational study have established that influenza infection can be associated with asthma exacerbation .But influenza vaccination itself has the potential to adversely affect pulmonary function. This study sought to determine whether influenza vaccination status is associated with asthma control in children or not.

## Methods

A survey was conducted of patients ≥6 yrs. old with a diagnosis of asthma who were registered in Iranian Pediatric Asthma Registration program (IPAR) in Immunology,Asthma and Allergy Research Institute (IAARI), Tehran, Iran since 2008. Mangement practice and level of asthma control, according to GINA guidline, for 517 children were evaluated. Data were analyzed using the statistical package SPSS version 16. Univarite analysis were performed to identify association between influenza vaccination and asthma control.

## Results

Among 172 well controlled patients, 59 one (34.3%) influenza vaccination was not current, 103 (59.54%) vaccination was current, and 10 patients didn't receive vaccine. In group II (partly controlled+ uncontrolled) 301 patients were registered, in which 164 (34.55%) was current, 131 (43.52%) was not current, and 64 (21.26%) didn't receive vaccine. There was a significant relation between receiving vaccination and well-controlled asthma (p<.000).

## Conclusions

Controversy exists regarding the effectiveness of influenza vaccination in improving asthma control in pediatric population. In this study it has been shown; using yearly influenza vaccination is related to better asthma control level in children and it's recommended to vaccinate all children with asthma.

